# Neighborhood disadvantage, frailty, and cognitive performance approaching midlife in the Colorado Adoption/Twin Study of Lifespan behavioral development and cognitive aging (CATSLife)

**DOI:** 10.1002/alz70860_100642

**Published:** 2025-12-23

**Authors:** Chandra A. Reynolds, Elizabeth Muñoz, Anqing Zheng, Ryan Bruellman, Robin P Corley, Soo H Rhee, Sally J Wadsworth, The CATSLife Team

**Affiliations:** ^1^ University of Colorado Boulder, Boulder, CO, USA; ^2^ University of Texas at Austin, Austin, TX, USA; ^3^ University of California Riverside, Riverside, CA, USA

## Abstract

**Background:**

Frailty is associated with poorer cognitive functioning and both have been associated with neighborhood deprivation. Whether such associations are apparent before midlife may provide a perspective on whether and how neighborhoods serve as a risk or resilience factor. The present study tested whether the association between neighborhood deprivation and cognitive functioning is mediated by frailty.

**Methods:**

We analyzed data from 1163 CATSLife participants (*M*age = 33 years, range 28‐49; 53% female, 10% non‐white/Hispanic; tested between 2015‐2021) with available age 16 IQ assessments. A general cognitive ability (GCA) factor was extracted from nine specific cognitive ability tests assessed by CATSLife and t‐score scaled. A 30‐item frailty index (FI), previously associated with processing speed, was included. A 2010 Area Deprivation Index (ADI) measure was constructed from seven census features (i.e., income, high school education, owner‐occupied units, poverty, unemployment, contract rent, female‐headed households) using a decile approach and averaged. FI and ADI were z‐scored before analysis. In path analysis, we regressed GCA on FI, and both were regressed on ADI, adjusting for adolescent IQ, age, sex, race/ethnicity, adoption status, and sibling similarity. We replicated findings with unranked 2018 Social Vulnerability Index SES scores (SVI‐SES) and WAIS‐III IQ scores.

**Results:**

Higher 2010 ADI was associated with higher FI (B=0.139, *p* <0.001) and lower GCA (B=‐0.426, *p* = 0.042), and higher FI was associated with lower GCA performance (B = ‐0.588, *p* = .011). The indirect effect between 2010 ADI and GCA via FI was significant (Indirect Estimate = ‐0.082, *p* = 0.031). Similar patterns were observed with the 2018 SVI‐SES, for FI and GCA (Indirect Estimate=‐0.126, *p* = 0.026), FI and full‐scale IQ (Indirect Estimate=‐0.098, *p* = 0.069), and FI and performance IQ (Indirect Estimate=‐0.160, *p* = 0.033), albeit not always achieving statistical significance.

**Conclusion:**

Adjusting for adolescent IQ, we observed associations of neighborhood disadvantage with lower general cognitive ability performance at midlife that may be connected via shared associations with frailty. Future work will expand to health behaviors (e.g., brain care scores) and further test temporality and selection factors.

